# Editorial: Endocrine regulation of aging: impacts of humoral factors and circulating mediators

**DOI:** 10.3389/fendo.2024.1387435

**Published:** 2024-03-06

**Authors:** Benjamin Petersen, Sharon Negri, Madison Milan, Zeke Reyff, Cade Ballard, Jennifer Ihuoma, Zoltan Ungvari, Stefano Tarantini

**Affiliations:** ^1^ Oklahoma Center for Geroscience and Healthy Brain Aging, University of Oklahoma Health Sciences Center, Oklahoma City, OK, United States; ^2^ Vascular Cognitive Impairment and Neurodegeneration Program, Department of Neurosurgery, University of Oklahoma Health Sciences Center, Oklahoma City, OK, United States; ^3^ International Training Program in Geroscience, Doctoral School of Basic and Translational Medicine/Department of Public Health, Semmelweis University, Budapest, Hungary; ^4^ Peggy and Charles Stephenson Cancer Center, University of Oklahoma Health Sciences Center, Oklahoma City, OK, United States; ^5^ Hudson College of Public Health, University of Oklahoma Health Sciences Center, Oklahoma City, OK, United States

**Keywords:** healthspan and lifespan, age-related disease, VCID, therapeutic targets, molecular mechanism

The intersection of endocrine regulation and aging merges two intricate and multi-dimensional fields of research, a convergence that is crucial in unraveling the biological mechanisms at the heart of aging. This synergy is pivotal in decoding the complexities of how hormonal balances and shifts influence the aging process, offering essential insights into the fundamental underpinnings of longevity and age-associated diseases (1–3). The focus of the Research Topic “*Endocrine Regulation of Aging: Impacts of Humoral Factors and Circulating Mediators*” is on highlighting the critical role of hormonal and circulating factors in influencing the aging process (4–6). This editorial compiles novel research articles that investigate the diverse roles of endocrine pathways, humoral factors, and circulating mediators in modulating the physiological aspects of aging. These studies contribute to our knowledge by clarifying molecular mechanisms, identifying potential therapeutic targets, and providing insights into the relationship between endocrine regulation and aging. This body of work aims to deepen our comprehension of the mechanisms by which endocrine factors influence lifespan and healthspan, setting a foundation for future research in this critical area of gerontology and endocrinology.

In an original article, Yang et al. evaluated the association between whole-blood nicotinamide adenine dinucleotide (NAD+) levels and the aging phenotype. The study included 1,518 participants in a large-scale community-based population survey. Each participant completed a survey and donated blood for analysis of NAD+ content. The results indicated that NAD+ levels decline with aging until age 50, with the most significant decrease observed in the 40–49 age group. Notably, this trend disappeared in participants over the age of 50. Gender comparison of NAD+ levels revealed a significant loss of whole-blood NAD+ content in middle-aged men, while levels in women were more variable, showing no significant differences among age groups. NAD+ has been the focal point of many studies on aging (7–10), but this study underscores the importance of considering gender differences in future investigations.

Jiao et al. investigated the relationship between metabolite levels and age-induced kidney dysfunction using a mouse model. The mice were divided into a young group (3 months) and an aged group (24 months), and metabolite abundance in kidney tissue and urine samples from the mice was compared via high-resolution untargeted metabolomics analysis. Aged mice exhibited inflammatory lesions, increased fibrosis, and functional impairment of the kidney. Metabolite analysis identified significant changes in metabolite concentrations of six key pathways involved in the metabolism of amino acids, carbohydrates, and nucleotides. These metabolite changes were linked to immune dysregulation, inflammation, oxidative stress, and cellular dysfunction exacerbating age-associated renal insufficiency. The study identified multiple metabolite biomarkers, offering potential indicators for early detection of and intervention in age-induced kidney dysfunction.

Dysregulation of hormonal activity has also been implicated in aging and the onset of disease. Zhang et al. explored the association between follicle-stimulating hormone (FSH) levels and the risk of developing rheumatoid arthritis (RA). The study compared serum sex hormone levels in 79 female RA patients with those of 50 age-matched controls, revealing that sex hormones were lower and FSH levels were elevated in RA patients. High FSH levels remained significantly associated with RA after adjusting for age and for luteinizing hormone, estradiol, and testosterone levels. Additionally, RA patients in the highest quartile of FSH levels exhibited increased disease burden. Sub-group analysis indicated a correlation between early menopause and age of onset of RA. Ultimately, these findings provide new insights into the role of age-related hormonal changes in the pathogenesis of RA and suggest FSH levels as a potential target for RA therapy and prevention.

Bickel et al. reviewed the mechanistic role of the somatotropic axis, specifically growth hormone (GH) and insulin-like growth factor 1 (IGF-1), on vascular aging and vascular cognitive impairment and dementia (VCID). The review reinforces recent results suggesting that cell non-autonomous mechanisms are a driving force in the cerebromicrovascular functional and structural changes associated with VCID. The findings highlighted the role of age-related GH/IGF-1 deficiency in cognitive impairment, exploring the macrovascular and microvascular adaptations linked to capillary rarefaction, microhemorrhages, impaired endothelial regulation, disruption of the blood–brain barrier, decreased neurovascular coupling, and atherogenesis. Understanding downstream molecular mediators of GH/IGF-1 is critical for the development of potential targeted interventions to promote cognitive health in older adults.

In a review examining pituitary adenylate cyclase-activating polypeptide (PACAP), Toth et al. explored the new and existing literature on the utility of PACAP as a diagnostic and prognostic indicator for age-related disease. The review presented results from *in vivo* and *in vitro* studies showing alterations of PACAP tissue levels and receptor expression in age-related diseases such as cerebrovascular disease, neurodegenerative disease, migraine, traumatic brain injury, post-traumatic stress disorder, chronic hepatitis, and nephrotic syndrome. The review discussed how animal models have been established to create PACAP knockout species, and many of the papers included show that PACAP-deficient animals exhibit age-related degenerative signs earlier. The review emphasizes the prevalence of PACAP in the body while recognizing the need for further research into the source of PACAP, as well as the anti-aging mechanism of the molecule within the human body.

In an original article, Ji et al. evaluated the influence of circulating galectin-3 (Gal-3) on the aging phenotype. Specifically, the study investigated the relationship between Gal-3 and frailty. Frailty is described as an age-associated decline in physiological function and resilience affecting multiple organ systems. The study included 149 elderly patients in a cross-sectional analysis; the findings revealed that frail participants, assessed based on the Fried frailty phenotype, exhibited higher serum Gal-3 concentrations compared to pre-frail and non-frail individuals. The findings from this cross-sectional study support existing evidence from mouse models that have previously been used to look at frailty and Gal-3 levels. Clinically, frailty is an indicator for poor health outcomes such as injury, prolonged hospitalization, and increased risk of mortality. The identification of a molecular indicator for frailty could provide a framework for important diagnostic and prognostic indicators for use in assessing health in the aging population.

Collectively, these papers underscore the intricate web of endocrine regulation, humoral factors, and circulating mediators in the aging process, offering novel insights into the mechanisms of aging and potential interventions. Not only does the highlighted research deepen our understanding of the complex biology of aging, but it also paves the way for future studies aiming to enhance the healthspan and mitigate age-associated diseases.

## Author contributions

BP: Writing – review & editing, Writing – original draft, Visualization, Validation, Supervision, Software, Resources, Project administration, Methodology, Investigation, Funding acquisition, Formal analysis, Data curation, Conceptualization. SN: Writing – review & editing, Writing – original draft, Visualization, Validation, Supervision, Software, Resources, Project administration, Methodology, Investigation, Funding acquisition, Formal analysis, Data curation, Conceptualization. MM: Writing – review & editing, Writing – original draft, Visualization, Validation, Supervision, Software, Resources, Project administration, Methodology, Investigation, Funding acquisition, Formal analysis, Data curation, Conceptualization. ZR: Writing – review & editing, Writing – original draft, Visualization, Validation, Supervision, Software, Resources, Project administration, Methodology, Investigation, Funding acquisition, Formal analysis, Data curation, Conceptualization. CB: Writing – review & editing, Writing – original draft, Visualization, Validation, Supervision, Software, Resources, Project administration, Methodology, Investigation, Funding acquisition, Formal analysis, Data curation, Conceptualization. JI: Writing – review & editing, Writing – original draft, Visualization, Validation, Supervision, Software, Resources, Project administration, Methodology, Investigation, Funding acquisition, Formal analysis, Data curation, Conceptualization. ZU: Writing – review & editing, Writing – original draft, Visualization, Validation, Supervision, Software, Resources, Project administration, Methodology, Investigation, Funding acquisition, Formal Analysis, Data curation, Conceptualization. ST: Writing – review & editing, Writing – original draft, Visualization, Validation, Supervision, Software, Resources, Project administration, Methodology, Investigation, Funding acquisition, Formal Analysis, Data curation, Conceptualization.
